# A Single-Center Real-World Experience: Early and Long-Term Outcomes of Pediatric Heart Transplantation with or Without a Left Ventricular Assist Device Bridging

**DOI:** 10.3390/jcm15031094

**Published:** 2026-01-30

**Authors:** Sedat Karaca, Ümit Kahraman, Osman Nuri Tuncer, Eser Doğan, Zülal Ülger Tutar, Yüksel Atay, Çağatay Engin, Tahir Yağdı, Mustafa Özbaran

**Affiliations:** 1 Department of Cardiovascular Surgery, Sanliurfa Training and Research Hospital, 63250 Sanliurfa, Turkey; 2 Department of Cardiovascular Surgery, Faculty of Medicine, Ege University, 35100 Izmir, Turkey; 3 Department of Pediatric Cardiology, Faculty of Medicine, Ege University, 35100 Izmir, Turkey

**Keywords:** pediatric heart failure, transplantation, LVAD, bridge, Htx

## Abstract

**Background**: Pediatric heart transplantation (HTx) is the standard therapy for end-stage heart failure in children, and the use of durable left ventricular assist devices (LVADs) as a bridge to transplant is increasing. However, comparative long-term data for LVAD-bridged versus directly transplanted pediatric recipients remain limited. In this study, we aimed to compare the early and long-term outcomes of pediatric heart transplantation with and without LVAD bridging. **Methods**: We retrospectively reviewed all pediatric patients who underwent orthotopic HTx at our institution between 2004 and 2024. 34 recipients were included, 17 bridged with durable LVAD support, and 17 transplanted without mechanical circulatory support. Perioperative characteristics, early postoperative complications, and long-term outcomes were compared between groups. **Results**: LVAD recipients had more advanced ventricular dysfunction, longer cardiopulmonary bypass and aortic cross-clamp times, and more frequent red blood cell transfusion requirements. Despite this higher-risk profile, early postoperative complications, early mortality, and ICU and hospital length of stay were similar between groups. Ten-year survival was 70.6% in the LVAD group, and 82.4% in the non-LVAD group (log-rank *p* = 0.365), and freedom from CAV and treated rejection did not differ significantly. **Conclusions**: In this single-center, two-decade experience, durable LVAD support enabled successful transplantation of high-risk pediatric candidates without compromising early or long-term post-transplant outcomes. LVAD bridging appears to be a safe and effective strategy in pediatric HTx.

## 1. Introduction

Pediatric heart transplantation (HTx) remains the gold standard therapy for children with end-stage heart failure, offering superior survival and quality of life compared with optimal medical therapy alone [1,2]. Despite its rarity, pediatric heart failure carries disproportionately high mortality rates [3], particularly in patients with complex congenital heart disease or cardiomyopathy. According to the International Society for Heart and Lung Transplantation (ISHLT) registry, median survival after pediatric HTx now exceeds 18 years, reflecting major advances in surgical techniques, perioperative care, and immunosuppressive regimens [4].

Over the past two decades, the use of mechanical circulatory support, particularly left ventricular assist devices (LVADs), has increased the rate at which children awaiting heart transplants benefit from this treatment. LVADs stabilize hemodynamics, preserve end-organ function, and increase the likelihood of reaching transplant [5,6]. International studies have demonstrated improved early post-transplant survival in LVAD-bridged recipients, concerns persist regarding infection, neurologic events, and the long-term durability of outcomes [7,8,9]. The pediatric experience with LVAD bridging remains more limited than in adults, largely due to patient size constraints and device availability. Despite encouraging results, the literature addressing long-term outcomes of pediatric HTx with versus without LVAD bridging remains sparse.

Beyond survival, pediatric heart transplantation outcomes are influenced by multiple factors, including pre-transplant clinical status, duration of wait-list time, and center-specific expertise [10]. Children bridged with LVAD support often represent a higher-risk cohort, characterized by advanced ventricular dysfunction, prolonged heart failure duration, and increased vulnerability to perioperative complications [11,12]. Moreover, LVAD implantation itself may introduce unique challenges, such as device-related infections, thromboembolic events, and sensitization, which can influence post-transplant morbidity and graft outcomes [11,12,13]. Consequently, the decision to implement LVAD support as a bridge to transplantation in pediatric patients remains complex and must balance the benefits of hemodynamic stabilization against potential device-related risks.

In this context, we sought to analyze our single-center, real-world experience with pediatric heart transplantation over the past two decades. Specifically, we compared short- and long-term outcomes between patients bridged with an LVAD and those transplanted without mechanical support, with the aim of clarifying the impact of LVAD bridging on survival, complication profiles, and overall clinical trajectory.

## 2. Materials and Methods

### 2.1. Study Design and Patient Population

This retrospective single-center study included all pediatric patients (<18 years) who underwent orthotopic heart transplantation at our institution between January 2004 and December 2024. A total of 34 patients were identified; 17 underwent direct HTx, and 17 were bridged with LVAD support. Exclusion criteria included patients aged ≥18 years and those transplanted at an outside institution but followed at our center. Patients were stratified into two groups: those bridged with durable LVAD prior to transplantation and those transplanted without mechanical circulatory support.

### 2.2. Data Collection and Variables

The following data were collected: recipient demographics, etiology of heart failure, history of LVAD implantation, preoperative echocardiographic parameters (left ventricular ejection fraction [LVEF], left atrial diameter [LA], left ventricular end-systolic and end-diastolic diameters [LVESD, LVEDD], severity of mitral and tricuspid regurgitation, and systolic pulmonary artery pressure), and preoperative laboratory findings (leukocyte count, hemoglobin, hematocrit, platelet count, alanine aminotransferase [ALT], aspartate aminotransferase [AST], albumin, urea, creatinine). Donor demographic data, preoperative immunosuppression regimen, intraoperative findings (cardiopulmonary bypass time, aortic cross-clamp time, cardiac ischemia time, need for pacing or inotropic support at weaning), and postoperative data (right ventricular failure, infection, acute kidney injury, respiratory complications, reoperation for bleeding, primary graft dysfunction (PGD), neurological complications, and mortality) were also recorded. Definitions of postoperative complications presented at Table 1.

### 2.3. Surgical Technique

All heart transplants were performed through a median sternotomy using the orthotopic biatrial technique, which represents the standard approach at our institution throughout the study period. Standard cardiopulmonary bypass was established with moderate hypothermia (28–32 °C). Myocardial protection for the donor heart was achieved by administering cold crystalloid cardioplegia immediately after aortic cross-clamping during donor procurement.

The recipient heart was completely excised, preserving adequate left and right atrial cuffs. The donor heart was implanted using continuous sutures for all anastomoses. The left and right atria were anastomosed as atrial cuffs, while the pulmonary artery and aorta were anastomosed separately in a standard end-to-end fashion.

In patients bridged with LVAD, surgery was initiated earlier to allow safe device explantation and meticulous hemostasis. Donor retrieval and recipient preparation were synchronized to minimize total ischemic time, ensuring that the donor heart arrived immediately prior to implantation for the shortest possible cold ischemia duration.

### 2.4. Perioperative and Postoperative Management

Perioperative management was standardized across all patients. Inotropic support was initiated after weaning from cardiopulmonary bypass using dopamine, dobutamine, epinephrine, and norepinephrine according to hemodynamic needs. Inhaled nitric oxide was administered in cases of elevated pulmonary vascular resistance or right ventricular dysfunction. Temporary pacing was used when indicated for bradyarrhythmias or conduction abnormalities. ECMO systems were kept on standby for potential intraoperative support; however, no patient required ECMO during the intraoperative period.

Postoperative rejection surveillance consisted of routine endomyocardial biopsies performed at 1, 3, 6, and 12 months after transplantation, and annually thereafter. Echocardiography was performed daily during the early postoperative period, monthly for the first 3 months, every 3 months thereafter, and annually after the first year. Rejection was graded according to the International Society for Heart and Lung Transplantation (ISHLT) criteria [14]. Infection prophylaxis was administered according to institutional protocols. LVAD-bridged patients were managed with device-specific anticoagulation and supportive care until transplantation. Cardiac allograft vasculopathy surveillance was not performed routinely as part of a standardized institutional screening program. Instead, coronary angiography was selectively performed in patients who developed chest pain, ischemic symptoms, graft dysfunction, or other clinical findings suggestive of coronary involvement. Imaging was obtained using conventional coronary angiography. For the purpose of this retrospective analysis, all available angiographic and clinical data were reviewed, and the diagnosis of cardiac allograft vasculopathy was reassessed in accordance with the most recent International Society for Heart and Lung Transplantation guidelines to ensure diagnostic consistency across the study period.

**Table 1:** Definitions of Postoperative Complications.

Complication	Definition
Right Ventricular Failure (RVF)	Need for prolonged inotropic or mechanical circulatory support (RVAD or ECMO) for ≥14 days post-transplant, or evidence of elevated central venous pressure with low cardiac output in the absence of left ventricular dysfunction.
Acute Kidney Injury (AKI)	Increase in serum creatinine ≥ 1.5× baseline within 7 days or urine output < 0.5 mL/kg/h for ≥6 h, in line with KDIGO criteria [15].
Respiratory	Prolonged mechanical ventilation (>48 h), reintubation, tracheostomy, or radiographically confirmed pneumonia with compatible clinical findings.
Primary Graft Dysfunction (PGD)	New-onset ventricular dysfunction within 24 h of transplantation not explained by rejection or surgical issues.
Graft Failure	Late-onset allograft dysfunction occurring beyond the early postoperative period, characterized by progressive ventricular dysfunction and/or hemodynamic compromise requiring inotropic support, mechanical circulatory support, or resulting in graft-related mortality, in the absence of acute rejection or surgical complications.
Cardiac Allograft Vasculopathy (CAV)	Presence of diffuse or focal coronary artery stenosis detected by coronary angiography or other imaging modalities during follow-up.
Infection	Clinically or microbiologically confirmed infection requiring targeted antimicrobial therapy during hospitalization.
Neurological	New postoperative stroke, transient ischemic attack, or encephalopathy confirmed by neurologic evaluation or imaging.

### 2.5. Immunosuppressive Therapy

All patients received a center-specific immunosuppressive regimen consisting of mycophenolate mofetil (MMF) and corticosteroids as maintenance therapy. Induction therapy included anti-thymocyte globulin (ATG) or an interleukin-2 receptor antagonist in selected patients. Cyclosporine was used in a minority of patients during the early study period, while tacrolimus subsequently became the standard calcineurin inhibitor in later years.

### 2.6. Statistical Analysis

All statistical analyses were performed using SPSS software (IBM Corp., version 25.0, USA). Descriptive variables were expressed as mean ± standard deviation (SD), median (interquartile range, IQR), or counts and percentages, as appropriate. The normality of distribution was assessed using the Shapiro–Wilk and Kolmogorov–Smirnov tests. Normally distributed continuous variables were compared using the independent-samples t-test, whereas non-normally distributed variables were analyzed using the Mann–Whitney U test. Categorical variables were compared using Fisher’s exact or chi-square tests, as appropriate.

Survival analyses were performed using the Kaplan–Meier method, and intergroup differences were assessed using the log-rank test. A two-tailed *p*-value < 0.05 was considered statistically significant.

## 3. Results

### 3.1. Baseline Characteristics

A total of 34 pediatric patients who underwent heart transplantation were included, with 17 (50%) bridged to transplant with an LVAD and 17 (50%) transplanted directly without prior mechanical circulatory support. The baseline demographic and clinical characteristics of both groups are summarized in Table 2.

Echocardiographic parameters demonstrated lower preoperative left ventricular ejection fractions in LVAD patients (median LVEF 19% vs. 23%, *p* = 0.031), consistent with more advanced ventricular failure before transplant. No intergroup differences were observed in atrial or ventricular dimensions, pulmonary artery pressure, or valvular regurgitation severity.

Preoperative laboratory values were largely comparable between groups. However, patients bridged with LVAD had significantly higher preoperative INR values (median 2.5 [2.1–3.0] vs. 1.0 [0.9–1.4], *p* < 0.001), reflecting ongoing anticoagulation management before transplantation. Liver function markers (AST, ALT, total bilirubin) and renal function indices (urea, creatinine) did not differ significantly.

All patients in both groups received a preoperative immunosuppressive regimen consisting of mycophenolate mofetil and corticosteroids according to institutional protocol.

### 3.2. Intraoperative Data

Intraoperative findings are summarized in Table 3.

Cardiopulmonary bypass and aortic cross-clamp durations were significantly longer in patients bridged with LVAD compared to those who underwent direct transplantation, likely reflecting the increased technical complexity associated with prior cardiac surgery (CPB time: 119 [108–152] vs. 95 [80–135] min, *p* = 0.015; XCL time: 88 [80–96] vs. 75 [61–90] min, *p* = 0.028).

Regarding transfusion requirements, LVAD recipients required a greater number of packed red blood cell (PRBC) units than non-LVAD patients (2 [1–3] vs. 1 [0–2] units, *p* = 0.03), while the amounts of fresh frozen plasma (FFP) and platelet transfusions were similar between groups (*p* = 0.213 and *p* = 0.124, respectively).

No patient in either group required intraoperative mechanical circulatory support, and all transplants were completed without conversion to ECMO assistance.

### 3.3. Early Postoperative Outcomes

Compared with primary transplant recipients, patients bridged with LVAD had numerically higher rates of several early postoperative complications; however, no statistically significant differences were observed between groups. The distribution of early morbidity and mortality parameters is summarized in Table 4.

Right ventricular failure, acute kidney injury, and respiratory complications occurred more frequently in the LVAD group, although these differences did not reach statistical significance. Primary graft dysfunction was observed in 1 (5.9%) patient in the non-LVAD group and in 2 (11.8%) patients in the LVAD group (*p* = 1.00). Postoperative ECMO support was required in 1 (5.9%) non-LVAD patient and 3 (17.6%) LVAD patients (*p* = 0.601).

Rates of infectious and neurological complications did not differ significantly between groups. The mean intensive care unit stay was 11.35 ± 6.23 days in the non-LVAD group and 12.06 ± 6.50 days in the LVAD group (*p* = 0.749). Median hospital length of stay was 32 days in both groups (IQR 26–46 vs. 26–48; *p* = 1.00).

Early (≤30-day or in-hospital) mortality occurred in 1 (5.9%) patient in the non-LVAD group and 3 (17.6%) patients in the LVAD group (*p* = 0.601). Given the limited sample size, these findings represent unadjusted descriptive comparisons rather than evidence of causal associations.

### 3.4. Long-Term Outcomes and Survival Analysis

**Figure 1:**
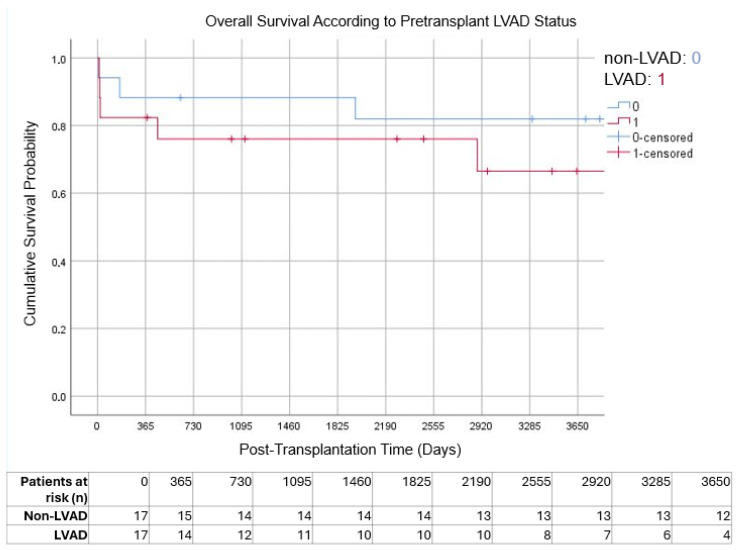
Kaplan–Meier curves demonstrating overall survival after pediatric heart transplantation according to pretransplant LVAD status. Numbers at risk are shown at yearly intervals below the x-axis.

During long-term follow-up, no statistically significant differences in overall survival were observed between patients bridged with LVAD support and those who underwent primary transplantation without prior device implantation. The estimated 1-, 5-, and 10-year survival rates were 82.4%, 76.5%, and 70.6% in the LVAD group and 88.3%, 88.3%, and 82.4% in the non-LVAD group, respectively (log-rank *p* = 0.365). Although numerically lower survival was observed among LVAD-bridged patients beyond five years of follow-up, this difference did not reach statistical significance (Figure 1).

**Figure 2:**
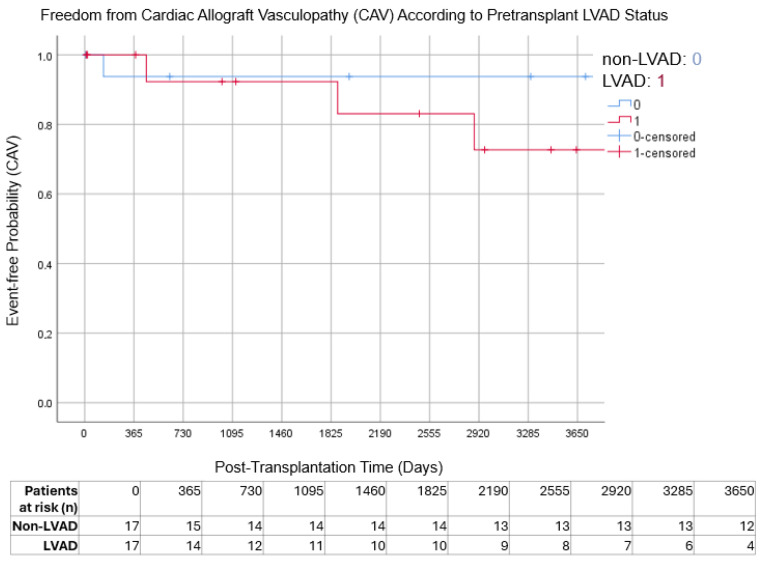
Freedom from cardiac allograft vasculopathy (CAV) according to pretransplant LVAD status. Numbers at risk are shown at yearly intervals below the x-axis.

**Figure 3:**
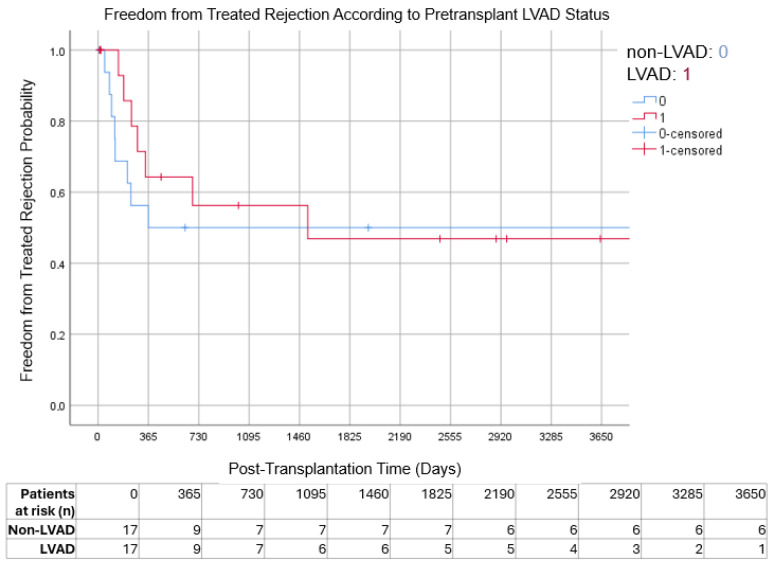
Kaplan–Meier analysis of freedom from treated rejection according to pretransplant LVAD status. Numbers at risk are shown at yearly intervals below the x-axis.

Freedom from cardiac allograft vasculopathy (CAV) and freedom from treated rejection also did not differ significantly between groups. Ten-year freedom from cardiac allograft vasculopathy was 76.5% in the LVAD group and 88.2% in the non-LVAD group (*p* = 0.144), while freedom from treated rejection at 10 years was 58.8% and 52.9%, respectively (*p* = 1.000) (Figure 2 and Figure 3).

Late post-transplant complications, including chronic kidney injury and malignancy, were observed at similar frequencies in both cohorts. Chronic kidney injury occurred in 23.5% of LVAD recipients and 17.6% of non-LVAD recipients, while malignancy was diagnosed in 5.9% of patients in each group. All patients who developed graft failure subsequently died during follow-up, and no cases of isolated graft dysfunction with long-term survival were identified.

## 4. Discussion

Our single-center real-world experience demonstrates that pediatric heart transplantation provides excellent short- and long-term survival, both in patients undergoing direct transplantation and in those bridged with durable LVAD support. Over a follow-up period extending beyond ten years, overall survival remained high in both groups, with no statistically significant differences observed, consistent with contemporary international registry data and international studies [7,16,17,18]. These findings are particularly relevant in pediatric heart failure, a population characterized by the highest pretransplant mortality among solid-organ transplant recipients [19].

Mechanical circulatory support has fundamentally altered the natural history of end-stage heart failure in children by stabilizing end-organ perfusion and enabling survival to transplantation, with similar observations also reported by Amdani et al. and Riggs et al. in their respective studies [8,20]. In our cohort, LVAD-bridged patients exhibited more advanced pretransplant ventricular dysfunction, with significantly lower left ventricular ejection fractions, confirming that device support was preferentially applied to the sickest candidates. Despite this unfavorable baseline profile, no statistically significant differences in early or long-term survival were observed compared with non-LVAD recipients. These observations support the clinical feasibility of LVAD support as a bridge to transplantation in high-risk pediatric candidates, rather than its use as a short-term rescue strategy [5,7,21].

Etiological differences between groups were also evident. Restrictive cardiomyopathy was observed exclusively in the non-LVAD cohort, whereas all LVAD recipients had dilated cardiomyopathy. Consistent with international reports, cardiomyopathies represent the most common underlying indication for pediatric heart transplantation [22,23,24]. An important consideration in the interpretation of our findings is the inherent selection bias arising from the suitability of LVAD support primarily in patients with dilated cardiomyopathy. Restrictive cardiomyopathy was observed only in the non-LVAD group, a distribution that reflects real-world clinical practice. Restrictive physiology is generally incompatible with durable LVAD support due to limited ventricular cavity size and, moreover, is characterized predominantly by a diastolic rather than systolic dysfunction pattern. In this context, the underlying cardiomyopathy subtype itself may independently influence both early and long-term post-transplant outcomes, irrespective of pretransplant mechanical circulatory support strategy. Therefore, although our findings support the safety of LVAD bridging, the observed results should be interpreted with appropriate caution.

Chronic anticoagulation during LVAD support resulted in significantly higher preoperative INR levels in the LVAD group and was accompanied by increased intraoperative packed red blood cell transfusion requirements. These findings highlight the persistent hemostatic challenges associated with device explantation and redo surgery. In the study by Rosenthal et al., no significant differences were identified between ischemic time and aortic cross-clamp duration [7]. However, in our study, cardiopulmonary bypass and aortic cross-clamp durations were longer in the LVAD group, whereas ischemic time, perioperative mortality, and early postoperative morbidity remained comparable between groups. This suggests that with meticulous surgical planning, synchronized donor–recipient operation planning, and experienced perioperative management, the technical complexity associated with LVAD explantation can be managed without an apparent increase in early procedural morbidity.

Early postoperative complications (right ventricular failure, acute kidney injury, respiratory complications, bleeding, infection, and neurological events) were numerically more frequent in the LVAD group but did not differ statistical significance between groups, and early postoperative mortality remained low in both cohorts. Long-term follow-up demonstrated sustained transplant outcomes in both groups; although a mild numerical trend toward lower survival beyond five years was observed among LVAD recipients, this difference was not statistically significant. Freedom from cardiac allograft vasculopathy and treated rejection likewise showed no statistically significant differences between groups. These findings suggest that pretransplant mechanical support does not confer a clear long-term disadvantage with respect to chronic graft vasculopathy or rejection burden, although continued lifelong surveillance remains mandatory in all pediatric recipients. In the study by Rosenthal et al., no significant differences were observed in postoperative ECMO use, graft failure, renal dysfunction, or neurological dysfunction [7]. Dipchand et al. reported a 5-year freedom from first rejection rate of 52%, while Kobayashi et al. documented a 10-year incidence of cardiac allograft vasculopathy of 25% [17,25]. Nevertheless, the overall survival observed in our cohort remains highly favorable and comparable to modern international benchmarks.

Because of the limited sample size and the small number of long-term deaths, we deliberately refrained from constructing a formal multivariable model for mortality. Nevertheless, we observed that severe postoperative complications, particularly the co-occurrence of ECMO requirement, acute kidney injury, and primary graft dysfunction were clustered almost exclusively among non-survivors. This pattern indicates that early multiorgan dysfunction represents a common final pathway leading to adverse outcomes after pediatric heart transplantation rather than isolated preoperative risk factors.

Over the two-decade study period, advances in imaging techniques and refinements in diagnostic criteria inevitably occurred. Nevertheless, core surveillance strategies for rejection, including routine endomyocardial biopsies and ISHLT-based grading systems, remained consistent [26]. Throughout the study period, our clinical approach was periodically revised and implemented in accordance with evolving international literature and guideline recommendations. All available imaging and clinical data were retrospectively reviewed and interpreted according to the most recent ISHLT guidelines to ensure diagnostic consistency across the study period.

Another important issue to be discussed is the progressive decline in transplant volumes, particularly in the pediatric population. Limited donor availability, advances in medical therapy and mechanical circulatory support that increase the number of patients surviving to transplantation, indicates that the demand for transplantable organs is unlikely to decrease in the coming years. In our study, 50% of patients were bridged to transplantation with an LVAD, whereas Voeller et al. reported a ventricular assist device utilization rate of only 6% in their 307-patient series published in 2013 [27]. These data suggest that the use of durable LVADs will continue to increase over time. Accordingly, there is an ongoing need for the development of novel devices specifically tailored for children with low body surface area.

Several limitations should be acknowledged. The retrospective nature of the study, the single-center design, and the small cohort size limit the generalizability of our findings and preclude definitive conclusions regarding independent risk factors for mortality. In particular, the limited sample size and low number of outcome events reduce the statistical power of the analyses and increase the risk of type II error, such that clinically relevant differences between groups may not have been detected. Accordingly, all analyses in this study were unadjusted and should be interpreted as descriptive rather than inferential, and no causal relationships can be established. Furthermore, heterogeneity in device platforms and immunosuppressive strategies over the two-decade study period may have introduced confounding, and the small number of events precluded multivariable adjustment. Nevertheless, the surgical approach and postoperative management were consistently maintained over a 20-year period by the same heart transplantation team at a single center, suggesting that the findings provide a meaningful real-world contribution to the existing literature and may serve as hypothesis-generating observations for future multicenter studies.

## 5. Conclusions

Taken together, our real-world two-decade experience demonstrates that pediatric heart transplantation achieves excellent early and long-term outcomes, irrespective of pretransplant LVAD support. Durable mechanical circulatory support enables successful transplantation in the sickest children. Therefore, pediatric patients with end-stage heart failure should be carefully evaluated for VAD implantation when appropriate. In the context of declining donor availability and the increasing number of children with advanced heart failure, optimized mechanical circulatory support strategies may facilitate improved outcomes and potentially enhanced survival in this vulnerable population. Larger multicenter and registry-based investigations are warranted to further refine patient selection criteria, optimize timing of LVAD implantation, and better define long-term outcomes. Such collaborative efforts may help inform evidence-based guidelines and support more individualized decision-making in pediatric heart transplantation.

## Figures and Tables

**Table 2:** Baseline characteristics of patients undergoing pediatric heart transplantation.

Variable	Non-LVAD (n = 17)	LVAD (n = 17)	*p*-Value
Demographics			
Age (years)	13 (7–17)	15 (11–16)	0.499
Sex, male n (%)	10 (58.8%)	10 (58.8%)	1.000
Height (cm)	143.4 ± 31.3	157.4 ± 19.1	0.127
Weight (kg)	39.0 ± 19.5	49.4 ± 15.6	0.096
Body surface area (m^2^)	1.24 ± 0.42	1.46 ± 0.31	0.102
Pretransplant waiting time (days)	384 (223–572)	271 (151–513)	0.235
**Etiology**			
Dilated cardiomyopathy n (%)	11 (64.7)	17 (100)	**0.018**
Restrictive cardiomyopathy n (%)	6 (35.3)	0 (0)	
Preoperative LVAD type n (%)	—	Heartware 9 (52.9%); EXCOR 7 (41.2%); HeartMate 3 1 (5.9%)	—
**Echocardiographic findings**			
LVEF (%)	23 (19.5–40.5)	19 (18–21.5)	**0.031**
LA diameter (cm)	2.28 ± 0.44	2.35 ± 0.38	0.651
LVESD (cm)	4.26 ± 1.73	5.24 ± 0.97	**0.041**
LVEDD (cm)	5.12 ± 1.45	5.85 ± 1.00	0.097
sPAP (mmHg)	25.7 ± 12.6	27.9 ± 11.8	0.597
Mitral regurgitation (≥moderate) n (%)	8 (47.1)	8 (47.1)	1.000
Tricuspid regurgitation (≥moderate) n (%)	7 (41.2)	8 (47.1)	0.910
**Laboratory findings**			
WBC (×10^3^/µL)	8.7 (7.7–13.0)	8.4 (6.1–11.0)	0.326
Hemoglobin (g/dL)	12.18 ± 2.17	11.62 ± 1.89	0.425
Platelet count (×10^3^/µL)	329.6 ± 82.2	353.8 ± 147.8	0.561
AST (U/L)	32 (24.5–66.0)	26 (22.5–46.0)	0.326
ALT (U/L)	28 (11.5–156.0)	23 (16–38.0)	0.605
Albumin (g/dL)	4.03 ± 0.71	4.06 ± 0.80	0.910
Total bilirubin (mg/dL)	1.33 ± 0.64	1.39 ± 0.53	0.786
INR	1.0 (0.9–1.4)	2.5 (2.1–3.0)	**<0.001**
Urea (mg/dL)	36 (27–40)	27 (20.5–43.5)	0.073
Creatinine (mg/dL)	0.66 (0.47–0.75)	0.64 (0.45–0.80)	0.730
**Preoperative immunosuppression n (%)** MMF + Corticosteroids	17 (100%)	17 (100%)	1.000

Notes: Data are presented as mean ± standard deviation (SD) for normally distributed variables; median (interquartile range, IQR) for non-normally distributed variables; and number (percentage) for categorical variables. Abbreviations: LVAD = left ventricular assist device; LVEF = left ventricular ejection fraction; LA = left atrium; LVESD = left ventricular end-systolic diameter; LVEDD = left ventricular end-diastolic diameter; sPAP = systolic pulmonary artery pressure; WBC = white blood cell count; AST = aspartate aminotransferase; ALT = alanine aminotransferase; INR = international normalized ratio; MMF = mycophenolate mofetil.

**Table 3:** Intraoperative data of pediatric heart transplant recipients.

Variable	Non-LVAD (n = 17)	LVAD (n = 17)	*p*-Value
CPB time (min)	95 (80–135)	119 (108–152)	**0.015**
XCL time (min)	75 (61–90)	88 (80–96)	**0.028**
Cardiac ischemia time (min)	210 (148–236)	185 (130–215)	0.408
PRBC transfused (units)	1 (0–2)	2 (1–3)	**0.030**
FFP transfused (units)	1 (1–2)	1 (1–2)	0.213
Platelet transfused (units)	0 (0–0)	0 (0–1)	0.124
Intraoperative pacing required, n (%)	9 (52.9%)	8 (47.1%)	1.000
Inotrope at CPB separation, n (%)	16 (94.1%)	17 (100%)	1.000
Intraoperative nitric oxide initiated, n (%)	6 (35.2%)	8 (47.1%)	0.728
Intraoperative ECMO used, n (%)	0 (0%)	0 (0%)	—

Notes: Data are presented as median (interquartile range, IQR) for continuous variables and number (percentage) for categorical variables. Cardiac ischemia time refers to the duration between donor aortic cross-clamp and graft reperfusion. Abbreviations: CPB = cardiopulmonary bypass; XCL = aortic cross-clamp; PRBC = packed red blood cells; FFP = fresh frozen plasma; ECMO = extracorporeal membrane oxygenation.

**Table 4:** Early Postoperative Outcomes.

Variable	Non-LVAD (n = 17)	LVAD (n = 17)	*p*-Value
Right ventricular failure, n (%)	1 (5.9%)	4 (23.5%)	0.335
Primary graft dysfunction, n (%)	1 (5.9%)	2 (11.8%)	1.000
Acute kidney injury, n (%)	3 (17.6%)	3 (17.6%)	1.000
Respiratory complications, n (%)	1 (5.9%)	4 (23.5%)	0.335
Reoperation for bleeding, n (%)	1 (5.9%)	3 (17.6%)	0.601
Infection (overall), n (%)	2 (11.8%)	2 (11.8%)	1.000
Neurological complications, n (%)	2 (11.8%)	4 (23.5%)	0.656
Postoperative ECMO support, n (%)	1 (5.9%)	3 (17.6%)	0.601
ICU stay (days), mean ± SD	11.35 ± 6.23	12.06 ± 6.50	0.749
Hospital stay (days), median (IQR)	32 (26–46)	32 (26–48)	1.000
Early mortality (≤30 days or in-hospital), n (%)	1 (5.9%)	3 (17.6%)	0.601

Data are presented as number (percentage) for categorical variables, mean ± standard deviation (SD) for normally distributed continuous variables, and median (interquartile range, IQR) for non-normally distributed continuous variables. Abbreviations: ECMO = extracorporeal membrane oxygenation; ICU = intensive care unit.

## Data Availability

The data presented in this study are available on request from the corresponding author. The data are not publicly available due to legal and regulatory restrictions.
